# Extra Physiotherapy in Critical Care (EPICC) Trial Protocol: a randomised controlled trial of intensive versus standard physical rehabilitation therapy in the critically ill

**DOI:** 10.1136/bmjopen-2015-008035

**Published:** 2015-05-25

**Authors:** Kirsty Thomas, Stephen E Wright, Gillian Watson, Catherine Baker, Victoria Stafford, Clare Wade, Thomas J Chadwick, Leigh Mansfield, Jennifer Wilkinson, Jing Shen, Mark Deverill, Stephen Bonner, Keith Hugill, Philip Howard, Andrea Henderson, Alistair Roy, Julie Furneval, Simon V Baudouin

**Affiliations:** 1 Newcastle upon Tyne Hospitals NHS Foundation Trust, Newcastle upon Tyne, UK; 2 Institute of Health and Society, Newcastle University, Newcastle upon Tyne, UK; 3 South Tees Hospitals NHS Foundation Trust, Middlesbrough, UK; 4 City Hospitals Sunderland NHS Foundation Trust, Sunderland, UK

**Keywords:** INTENSIVE & CRITICAL CARE, Physiotherapy

## Abstract

**Introduction:**

Patients discharged from Critical Care suffer from excessive longer term morbidity and mortality. Physical and mental health measures of quality of life show a marked and immediate fall after admission to Critical Care with some recovery over time. However, physical function is still significantly reduced at 6 months. The National Institute for Health and Care Excellence clinical guideline on rehabilitation after critical illness, identified the need for high-quality randomised controlled trials to determine the most effective rehabilitation strategy for critically ill patients at risk of critical illness-associated physical morbidity. In response to this, we will conduct a randomised controlled trial, comparing physiotherapy aimed at early and intensive patient mobilisation with routine care. We hypothesise that this intervention will improve physical outcomes and the mental health and functional well-being of survivors of critical illness.

**Methods and analysis:**

308 adult patients who have received more than 48 h of non-invasive or invasive ventilation in Critical Care will be recruited to a patient-randomised, parallel group, controlled trial, comparing two intensities of physiotherapy. Participants will be randomised to receive either standard or intensive physiotherapy for the duration of their Critical Care admission. Outcomes will be recorded on Critical Care discharge, at 3 and 6 months following initial recruitment to the study. The primary outcome measure is physical health at 6 months, as measured by the SF-36 Physical Component Summary. Secondary outcomes include assessment of mental health, activities of daily living, delirium and ventilator-free days. We will also include a health economic analysis.

**Ethics and dissemination:**

The trial has ethical approval from Newcastle and North Tyneside 2 Research Ethics Committee (11/NE/0206). There is a Trial Oversight Committee including an independent chair. The results of the study will be submitted for publication in peer-reviewed journals and presented at national and international scientific meetings.

**Trial registration number:**

ISRCTN20436833.

**Strengths and limitations of this study**

- The strengths of the study include the relatively large sample size and pretrial power calculation, the inclusion of a range of general Critical Care patients, the multicentre recruitment (although limited to three centres in the north of England), applicability to UK practice, delivery of rehabilitation to the control group; which reflects current UK practice, and prolonged (6 months) follow-up.
- Limitations include the number of sites and the lack of 7-day rehabilitation interventions. Also, the study is blinded to patient participants, but is not blinded to their healthcare providers.

## Introduction

### Background and rationale

Over 100 000 patients are admitted to Critical Care Units in the UK every year. It has been recognised for some time that patients discharged from Critical Care suffer from excessive longer term morbidity and mortality.^1^ Quality of life (QoL), in terms of both physical and mental health, is significantly reduced following a prolonged admission to Critical Care. In survivors, there is a slow and incomplete recovery in QoL over the next 6–12 months.^2^

In 2009, the National Institute for Health and Care Excellence (NICE) published guidance for rehabilitation after critical illness.^3^ The guideline noted that there were no published randomised controlled trials examining how effective early mobilisation therapy is at reducing the risk of adult patients developing physical and non-physical morbidity after hospital discharge. The authors recommended more research to determine which therapeutic strategies are the most clinical and cost effective at reducing the prevalence and severity of critical illness-associated physical morbidity, psychological morbidity, and cognitive dysfunction.

Since 2009, there have been a small number of published randomised controlled trials of mobilisation therapy in Critical Care. In a study based in two Medical Critical Care Units in North America, 104 patients who were previously functionally independent were randomised to receive either early mobilisation therapy or standard care. Early mobilisation therapy was found to be safe and well tolerated, and resulted in better functional outcomes at hospital discharge, a shorter duration of delirium, and more ventilator-free days compared with standard care.^4^ A single-centre study in medical and surgical Critical Care patients randomised patients to receive either daily standard physiotherapy or daily standard physiotherapy with an active training session using a bedside cycle ergometer. They found that additional exercise training enhanced recovery of functional exercise capacity, self-perceived functional status, and muscle force at hospital discharge.^5^ A single-centre randomised controlled trial in Australia compared normal physiotherapy (active exercises and progressive mobilisation, 6 days/week, until hospital discharge) to an intensive physiotherapy programme started in Critical Care and continued through the ward stay and after hospital discharge.^6^ There were no significant differences in any of the outcome measures at any stage of follow-up although the rate of change over time from first assessment was greater in the intervention group. Recent systematic reviews and meta-analyses identified the need for further controlled trials of better quality and larger sample size studies, including evaluation of type, duration, frequency and intensity of physical therapy.^7–10^

There is, therefore, some evidence that early physiotherapy in this group of severely ill patients may improve both the rate and magnitude of recovery from critical illness. However, the evidence base is incomplete and, in particular, the optimum intensity of physiotherapy is not known. To address this, we will carry out a randomised controlled trial of intensive versus routine physiotherapy in the critically ill.

### Objective

The objective of the study is to compare two different intensities of early physiotherapy on the rate and magnitude of recovery from critical illness in a general Critical Care population of adult patients.

### Study design

**Figure 1:**
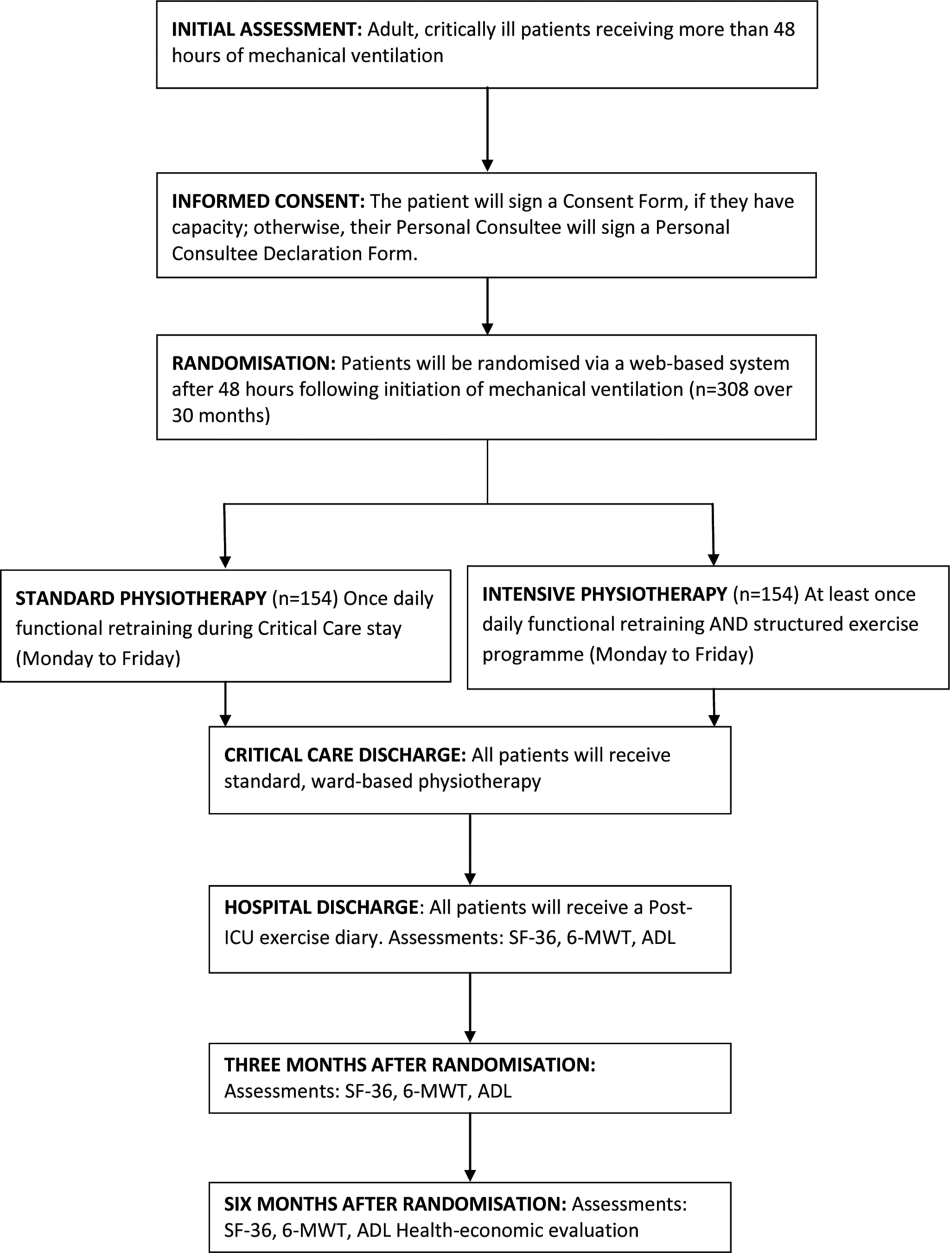
Study design.

We will conduct a multicentre patient randomised, parallel group, controlled trial comparing two intensities of physiotherapy on physical QoL. Adult, critically ill patients, ventilated for more than 48 h, will be randomised to the control or intervention group (figure 1). The control group will receive usual physiotherapy, including a once-daily functional retraining session (Monday to Friday). The intervention group will receive a more intensive physiotherapy programme, including at least one functional retraining session per day (Monday to Friday) and an individualised structured exercise programme.

## Methods

### Eligibility criteria

Participants will be recruited from the Critical Care (Intensive Care or High Dependency) Units of participating hospitals. Eligible patients will be medical or surgical patients aged 18 years or older having received 48 h or more of invasive or non-invasive mechanical ventilation. The exclusion criteria are as follows: patients receiving end-of-life care; patients with acute brain or spinal cord injury; patients admitted following brain or spinal cord surgery; patients with multiple trauma if mobilisation therapy is unlikely to be possible; patients with burns; patients with rapidly progressive neuromuscular disease; patients enrolled in another clinical trial without a co-enrolment agreement in place; patients previously enrolled in the Extra Physiotherapy in Critical Care study. Patients who have suffered cardiac arrest may be recruited if the clinical team believes that there is a possibility of recovery.

### Study intervention

**Figure 2:**
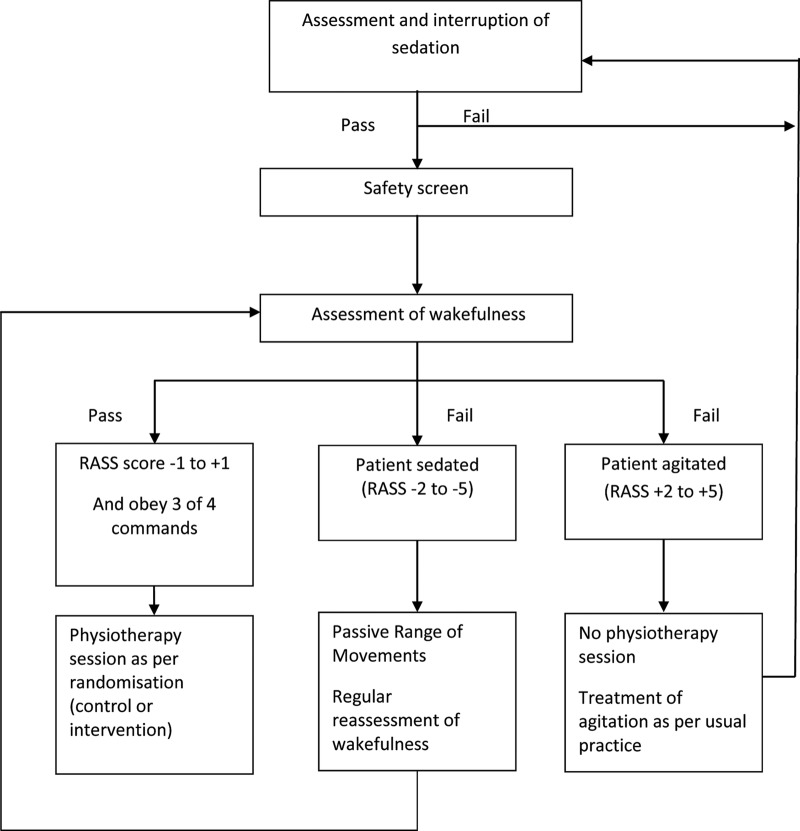
Trial intervention protocol.

Following randomisation into the trial, each participant will undergo a daily morning sedation hold, with the exception of those participants who have neuromuscular blocking agents administered, where severe agitation is already present despite sedation, or where participants’ ventilatory parameters are so high that a sedation hold would be deemed detrimental to participant safety. The sedation hold will consist of the interruption of all continuous intravenous sedation^11^ and assessment of the patient using the Richmond Agitation-Sedation Scale (RASS).^12^ Participants will be assessed continuously during this period, and sedation re-started (if required) to achieve a sedation level of ‘easily roused and cooperative’ (RASS of −1, 0 or +1). If the sedation hold is successful, the participant will be screened for safety prior to the start of physiotherapy (figure 2). If the participant fails the sedation hold, or a sedation hold is not appropriate, passive range of motion exercises will be performed in all four limbs and the trunk, as deemed appropriate. The assessment of sedation will be repeated for both groups in the afternoon with a second adjustment of sedation if needed. This will control for sedation breaks in that both groups will, on average, have had their sedation controlled in the same manner. Following this, the participants will be screened for safety and undertake physiotherapy as appropriate for the group to which they have been randomised.

The safety screen will be conducted by the treating physiotherapist in conjunction with nursing and medical staff, as necessary. Participants meeting any of the following criteria will fail the safety screen: mean arterial pressure (MAP) <65 mm Hg; heart rate <40 or >130 bpm; respiratory rate <5 or >40 breaths/min; marked ventilator asynchrony; oxygen saturation <88% or, if known respiratory disease, a 10% decrease from normal oxygen saturation; active gastrointestinal bleeding; acute myocardial ischaemia; actively undergoing a procedure; agitation requiring increased sedatives in the last 30 min; insecure airway; other significant events deemed inappropriate by the Critical Care team for the start of active physiotherapy (specifics to be recorded).

Following a successful safety screen, an assessment for wakefulness will be completed. The participant must be able to obey at least three of the following commands:^11^ (1) opening eyes in response to voice; (2)using eyes to follow investigator on request; (3)squeezing hand on request and (4)protruding tongue on request. If the participant can follow at least three of these commands and has a RASS of −1, 0 or +1, they will be deemed as having achieved ‘wakefulness’, and active physiotherapy can start (figure 2).

**Figure 3:**
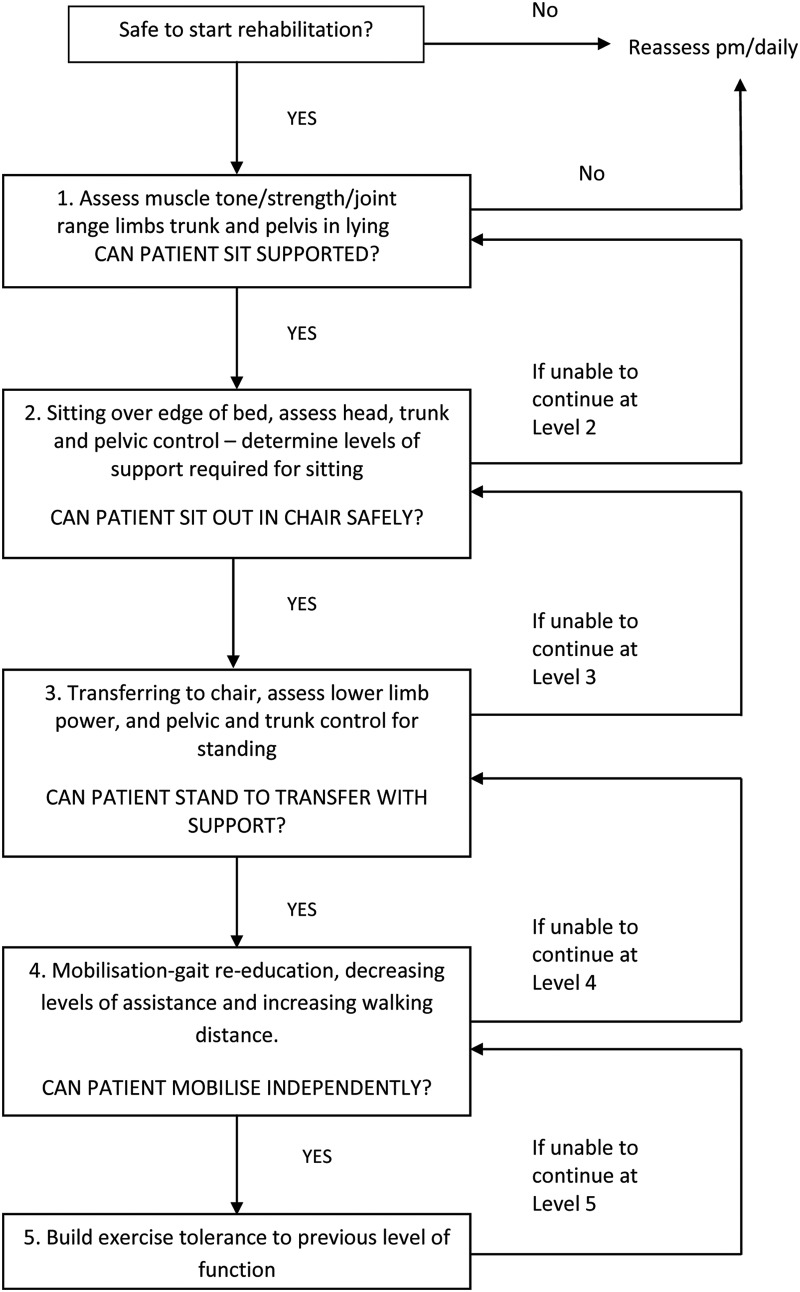
Functional retraining flow chart.

**Figure 4:**
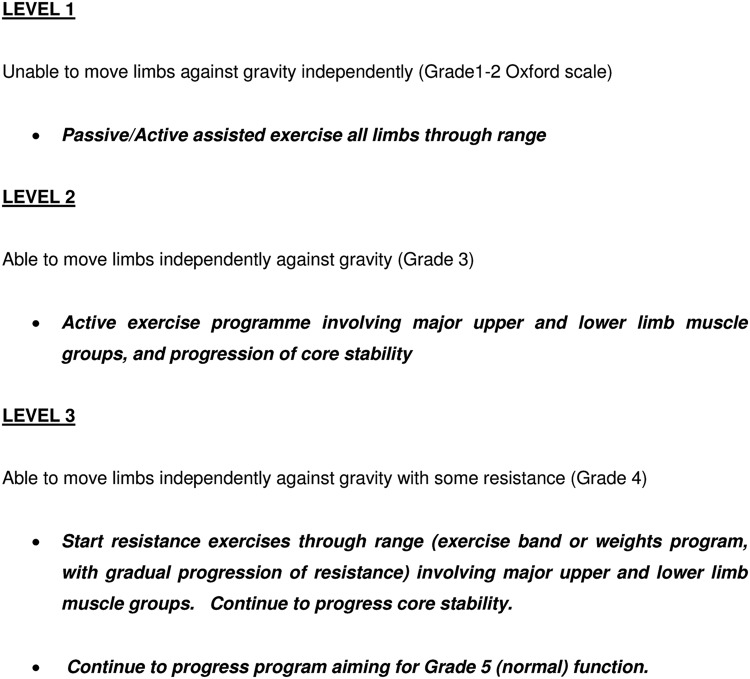
Strengthening programme algorithm.

The physiotherapy techniques used in this trial include functional retraining (figure 3) and individually tailored exercise programmes, based on a muscle-strengthening algorithm (figure 4). During functional retraining, participants will undergo physiotherapy at one of five levels of activity, only moving onto the next level when they are able to achieve the activity at the current level. During the individually tailored exercise programmes, participants will progress from active-assisted exercises through to resisted exercises, via the use of various therapy adjuncts, that is, hand weights, exercise band, and computer game-based exercise. The duration and content of therapy sessions from both treatment groups will be recorded daily.

The control group will receive the standard intensity of physiotherapy which is normally given on participating units. This will consist of no more than 30 min of active exercise comprising: functional retraining (figure 3) and, if time permits, an individually tailored exercise programme, based on a muscle-strengthening algorithm (figure 4). Physiotherapy will occur from Monday to Friday as there is no physiotherapy service available routinely at weekends.

The intervention group will receive an augmented programme with a target delivery of 90 min of active exercise per day, again from Monday to Friday. The physiotherapy sessions will consist of functional retraining (figure 3) and an individually tailored exercise programme, based on a muscle-strengthening algorithm (figure 4). The delivery of this treatment will be divided into one or more sessions depending on: the individual participant's tolerance of the programme; the need for other medical and nursing interventions; and the progression of the participant's recovery.

During the physiotherapy sessions, participants will be monitored closely, and if any of the following occur, the session will be stopped: MAP <60 mm Hg; heart rate <40 or >130 bpm; oxygen saturation <88% (or, if known respiratory disease, a 10% decrease from normal oxygen saturation); marked ventilator asynchrony; participant distress; new arrhythmias; myocardial ischaemia; concern for airway integrity; endotracheal tube removal; fall to knees; or any other significant event where the participant is unable to continue rehabilitation.

Apart from the difference in the intensity of physiotherapy, both groups will have the usual rehabilitation pathway in participating units.

Following discharge from Critical Care, both groups will receive the same, standard intensity of ward-based physiotherapy, and continue with their rehabilitation pathway. In line with NICE guidance, participants will receive an exercise diary to continue independently on discharge from hospital. Any patient readmitted to Critical Care will receive standard physiotherapy care during the second and any subsequent readmissions. All readmissions will be recorded.

### Outcomes

#### Primary outcome measure

The primary outcome measure is physical health as measured by the Short Form 36 Health Survey (SF-36) Physical Component Summary (PCS) score at 6 months after randomisation.

#### Secondary outcome measures

Mental health: the SF-36 Mental Component Summary (MCS) score.

Exercise capacity: the 6 min walk test.^13^

Muscle power: hand grip and quadriceps muscle strength using a dynamometer^14^ (see online supplementary appendix S1).

Physical functional ability: measured at Critical Care discharge using the Modified Rivermead Mobility Index.^15^

Activities of daily living (ADLs): ability to perform ADLs will be assessed using the Functional Independence Measure (FIM).^16^ The FIM and other questionnaires may be completed via a telephone call, rather than in person, at the 3-month and 6-month follow-up visits.

QoL: SF-36 and EQ-5D questionnaires will be administered to assess participants’ QoL. Utility values will be derived from the EQ-5D and from the SF-36 using the algorithm provided by the SF-6D.^17^ These will be used as the outcome measures in the economic analysis.

Outcome and location: survival status and place of residence will be recorded at hospital discharge, and 3 and 6 months following randomisation.

Delirium: before beginning rehabilitation, participants will be screened for delirium using the Confusion Assessment Method for the Intensive Care Unit.^18^

Ventilator-free days: defined as the number of days from randomisation (day 1) to day 28 on which a patient breathed without mechanical ventilation (includes non-invasive ventilation) if the period of unassisted breathing lasted at least 48 consecutive hours. Continuous positive airways pressure is considered to be unassisted breathing.

Health economic outcomes: utility scores will be generated using the SF-6D algorithm and the EQ-5D scores. Patient costs for hospital visits will be assessed at 6 months, using the Patient Costs Questionnaire (see online supplementary appendix S2).

The time schedule of assessments of participants is shown in table 1.

**Table 1:** Schedule of data collection and outcome assessments

	Randomisation	Rehabilitation sessions	Critical Care discharge	Hospital discharge	3 Months from enrolment	6 Months from enrolment
Demographic and diagnostic data	X					
Severity of illness: APACHE II score	X					
Premorbid activity	X					
Sedation hold		X				
Safety screen		X				
Session duration		X				
Session milestones		X				
Adverse events		X				
Quality of life: SF-36 and EQ-5D				X	X	X
Six-minute walk test				X	X	X
Functional Independence Measure			X	X	X	X
Grip strength, both hands			X	X	X	X
Quad strength, both legs			X	X	X	X
Body mass index			X	X	X	X
Modified Rivermead Mobility Index			X			
Patient costs questionnaire						X

APACHE, Acute Physiology and Chronic Health Evaluation; SF-36, Short Form 36 Health Survey.

### Sample size

The trial is powered to detect a difference of five points on our primary outcome (the PCS score of the SF-36 at 6 months following recruitment) between the intervention and control groups. In a previous study, we found that mean PCS at 6 months after hospital discharge was 34 with a SD of 10.^19^ This SD is similar to that reported at 6 months in 110 participants in the standard limb of the PRaCTICal UK 20 follow-up study (SD 11.7), and in a follow-up study of 252 critically ill patients in The Netherlands (SD 11.2).^21^ Taking a SD of 11, a five-point difference would give a medium effect size of 0.45. A sample size calculation based on a power of 80% to detect a five-point difference in SD at a significance level of 0.05 would require 77 participants in each group, a total of 154 for the trial. However, the 6-month mortality in this group of patients is known to be approximately 40%. In addition, we anticipate that we may lose a further 10% of participants to follow-up for other reasons. We will therefore need to recruit a total of 308 participants (154 in each group).

### Randomisation

Participants will be randomised in a 1:1 ratio, using permuted random block allocation, to either intervention or control group. The randomisation process will be administered centrally by Newcastle Clinical Trials Unit via a secure web-based system. Randomisation will be stratified by admitting Critical Care Unit, type of admission (surgical or medical) and the participant's prehospitalisation activity level. We will use Katz's ADL Index^22^ to stratify participants into either ‘low’ activity (score of 0–3) or ‘high’ activity (score of 4–6).

### Blinding

Owing to the nature of the intervention, it is not possible to blind the participants, clinical staff or research staff to the treatment group. However, the outcome assessments at hospital discharge, and at 3 and 6 months after randomisation, will be undertaken by a research nurse who will be blinded to the treatment group. To avoid accidental unblinding, patients attending for assessment will be requested not to reveal their treatment group to the research nurse.

### Data collection

The study data collected at each time point are summarised in table 1. All participating units contribute data to the Intensive Care National Audit and Research Centre (ICNARC) case mix programme. Data collected routinely by units as part of this national audit will be used to provide demographic, diagnostic and severity of illness data for participants. These data will include: age; gender; admission diagnoses; Acute Physiology and Chronic Health Evaluation (APACHE) II severity of illness score; number of organ failures; admission and discharge location; length of stay (in Critical Care and in hospital); status on hospital discharge; and ultimate discharge location. An estimate of each participant's premorbid functional ability (Katz's ADL Index) will be made by interview with their next of kin. For each day a participant remains in Critical Care, we will record the type and total dose of sedatives and the total calorie intake received as enteral or parenteral nutrition. Data related to each physiotherapy session will be collected by the physiotherapist allocated to that session, under the supervision of the research physiotherapist. We will assess participants at discharge from Critical Care against the Modified Rivermead Mobility Index. At hospital discharge, 3 and 6 months after study enrolment, patients will be invited to attend a follow-up appointment where the study assessments will take place. Where feasible, appointments will coincide with routine clinical follow-up, to enhance the likelihood of good compliance. Participants who do not attend their appointment will be followed up by telephone.

### Data management

To preserve confidentiality, all participants will be allocated a unique study identifier, which will be used on all case report forms and questionnaires. To allow for the possibility that the incorrect unique study identifier is recorded, the participant's initials will also be recorded on each case report form and questionnaire. Only a limited number of members of the research team will be able to link this identifier to patient-identifiable data, which will be held on a password-protected data base. All study documentation will be held in secure offices. Data management will be undertaken using a Microsoft Access database for data entry and processing, allowing a full audit trail of any alterations made to the data postentry. Original case report forms, questionnaires and consent forms will be archived securely at The Newcastle upon Tyne Hospitals NHS Foundation Trust's archive facility for 5 years following publication of the last paper or report from the study. Data will be handled, computerised and stored in accordance with the Data Protection Act 1998. No patient-identifiable data will leave the study sites. The quality and retention of study data will be the responsibility of the Chief Investigator. All study data will be retained in accordance with the latest Directive on Good Clinical Practice (2005/28/EC).

### Trial registration

In keeping with Good Clinical Practice, the study underwent all ethical requirements and applications for registration prior to the start of recruitment to the study. Application for registration of the trial via the National Institute for Health Research Integrated Research Application System was submitted on 30 June 2011, but due to a delay in administration, the trial did not appear on the registry until 20 February 2012, shortly after the start of recruitment. At this particular time, a system which automatically registered National Institute for Health Research-funded studies via the IRAS system was not in place. All legal ethical requirements were in place prior to the start of recruitment.

## Analysis

### Statistical analysis

The PCS component of the SF-36 at 6 months following recruitment is the primary end point of the study. In order to test the primary hypothesis of no difference between the intervention and control groups, the principal analysis will examine the difference between the groups on this measure using Analysis of Covariance (ANCOVA) in order to adjust for the effects of covariates in the analysis. The normality of the outcome variable will be assessed and, if necessary, transformations considered although ANCOVA are robust to deviations from this distribution. The covariates to be considered will include stratification variables (unit, admission type and preadmission activity level) in addition to demographic variables such as age and gender. More basic illustrative analyses using the t test or non-parametric alternatives may also be undertaken while, in addition, summary statistics will be calculated.

A broadly similar approach will be used to analyse the secondary outcome measures (the MCS component of the SF-36, the 6 min walk test, FIM, grip strength, quadriceps strength and body mass index (BMI)) at both 3 and 6 months post-study entry, in addition to at hospital discharge. Additionally, the 3-month and hospital discharge data on the PCS component of the SF-36 will be analysed in this fashion, along with the FIM, grip strength and BMI outcomes at Critical Care discharge. Repeated measures methods (again based on analysis of variance) will be employed in order to examine these outcome measures longitudinally across collected time points. Standard survival data methods, such as Kaplan-Meier and the log rank test, will be used to compare survival between the groups at 3 and 6 months. We will also compare durations of Critical Care and hospital stay, in addition to the delirium and ventilator-free days measures.

There are no planned interim analyses or stopping rules. An allowance for loss to follow-up has been included in the sample size calculation; data with missing observations due to loss to follow-up, as opposed to mortality, will be examined to determine both the extent of missing data and whether data are missing at random or are informative. If data are missing to a sufficient extent, the use of appropriate imputation techniques will be considered to allow for this in the analysis. All analyses will initially be performed on an intention-to-treat basis, although it may be useful to consider additional per-protocol analyses. We may base an analysis on groups defined by a dichotomisation (into long or short) length of stay in Critical Care, in addition to considering other subgroup analyses. A statistical analysis plan will be written and agreed by the Trial Oversight Committee (TOC) before analysis of the study data.

### Economic analysis

The economic analysis within this trial will consist of an extensive cost analysis of both the control and intervention groups from the NHS perspective, and will identify the key items (if any) that underpin any significant cost differences between the two trial groups. A societal perspective on costs will also be taken to include costs that fall on the patients, their carers/families and/or non-NHS cost centres that result from the different treatment regimes. Data will be gathered through the administration of the Participant Costs Questionnaire (see online supplementary appendix S2), which was designed specifically for this study, and may be the first of its type to be published. This latter work will complement the main NHS perspective of the cost analysis. In terms of effectiveness, health-related QoL data based on the SF-36 collected at three time points in the trial's follow-up will be analysed using the SF-6D algorithm^17^ to produce utility scores. Utility scores based on SF-36 will also be compared against absolute utility scores derived from the EQ-5D^23^ collected at the 6-month follow-up. Depending on what the data show, and what realistic assumptions we can make, we may proceed to a cost-utility analysis and undertake extensive probabilistic sensitivity analysis.^24^

### Trial management

Day-to-day running of the trial will be overseen by a TMG, comprising, as a minimum, the Chief Investigator, Senior Trial Manager, Trial Manager, Trial Statistician and Data Manager. TMG meetings will take place on a regular basis throughout the duration of the study. The TMG will have responsibility for ensuring the compliance and progress of the study in relation to all regulatory, administrative academic and any clinical or safety issues.

### Trial monitoring and oversight

Monitoring of study conduct and data collected will be performed by a combination of central review and site monitoring visits to ensure the study is conducted in accordance with the International Conference on Harmonisation Good Clinical Practice Guidelines. Study site monitoring will be undertaken by the Newcastle Clinical Trials Unit.

As agreed by the sponsor, a TOC will adopt the joint roles of Trial Steering Committee (TSC) and Data Monitoring and Ethics Committee (DMEC), with independent members meeting in a closed session to fulfil the DMEC role. The TOC will consist of the Chief Investigator, an independent Chair/clinician, the Trial Statistician, independent lay representative and the TMG; a representative of the funder will be invited to attend. The purpose of this committee will be to monitor efficacy and safety end points, although only independent members will have access to un-blinded study data. A written charter will be agreed and used by the TOC.

After 6 months of recruitment, initial rates of recruitment will be used to project total recruitment, to ensure sufficient participants to power the trial. The TOC will advise on whether to continue or discontinue the study and make a recommendation to the sponsor. The trial may be prematurely discontinued on the basis of new safety information, or for other reasons given by the TOC, sponsor, regulatory authority or research ethics committee. If the study is discontinued prematurely, active participants will be informed, and no further participant data will be collected.

### Adverse event reporting

The participants recruited into this study are critically ill with a high likelihood of significant morbidity and mortality. Many will have life-threatening conditions, and we expect that a large proportion of the participants will experience Adverse Events (AE) and Significant Adverse Events (SAE) during the time between randomisation and discharge from Critical Care. For this reason, the time period for AE and SAE reporting will begin with each sedation hold (if performed), continue through each physiotherapy session, and end 30 min after the end of each physiotherapy session. All adverse events occurring during the reporting period will be recorded on an AE/SAE Report Form, and categorised as to the degree of expectedness, relatedness and severity. All SAEs that occur during the reporting period must be reported to the Chief Investigator within 24 h of learning of the event, and a completed report form sent to the Trial Manager. An SAE occurring to a research participant should be reported to the main research ethics committee where, in the opinion of the Principal Investigator, the event was related to administration of any of the research procedures and unexpected (ie, not listed in the protocol as an expected occurrence). A confirmed, related SAE will be reported to the research ethics committee within 15 days of the Chief Investigator becoming aware of the event. In addition, the trial sponsor will be notified of the SAE.

There are a number of AEs that may be expected to occur in patients undergoing physiotherapy in Critical Care and do not need to be reported, these are: MAP <65 mm Hg; HR<40 or >130 bpm; respiratory rate <5 or >40 breaths/min; marked ventilator asynchrony; oxygen saturation <88% or, if known respiratory disease, a 10% decrease from normal oxygen saturation; marked ventilator asynchrony; patient distress; new cardiac arrhythmia; myocardial ischaemia; insecure airway; fall to knees.

## Ethics and dissemination

### Consent

The consent process will depend on whether the patient has capacity to give informed consent. Eligible patients with capacity to consent will be approached by trained members of the research team who will describe the trial, including the potential risks and benefits, and provide a participant information sheet. The patient will be given time to consider whether they wish to take part in the trial and to ask any questions. The member of the research team will then invite the patient to sign the consent form. If an eligible patient lacks capacity to consent, a personal consultee will be approached in the same way as for a patient with capacity. They will be asked whether, in their opinion, the patient would agree to take part in the trial, and will be invited to sign the personal consultee declaration form. If, despite reasonable efforts by the research team a personal consultee is not available, a professional consultee will be approached and given an information sheet. The professional consultee will be the consultant responsible for the patient's care before admission to Critical Care. In determining what the person's wishes and feelings about the research would be if they had capacity, the nominated professional consultee should attempt to seek views from any family, friends or carers who may not be willing or able to act as a personal consultee. Where appropriate, other professional colleagues with an interest in the welfare or condition of the person who lacks capacity, such as other members of the care team not involved in the research, may be approached for a view. If the professional consultee deems that the patient would be willing to enter the study, they will be invited to sign a professional consultee declaration form.

### Retrospective consent

If a participant recovers from their illness and regains capacity to give consent, the same consent procedure will be carried out, as though the participant had capacity before randomisation. In these cases, a retrospective patient information sheet and consent form will be used.

### Participants who lose the capacity to consent during the trial period

The majority of critically ill patients will suffer from periods of impaired consciousness where their capacity to make informed decisions will be impaired or absent. If a patient has consented to join the study, it will be assumed that he or she would wish to continue in the trial, even if the capacity to make an informed decision is subsequently lost.

We anticipate a relatively short time window between the time of patients meeting eligibility criteria and the time that physiotherapy would be feasible. Because of the need to give patients and personal consultees sufficient time to consider participation in the trial, we will provide information sheets for patients who are judged likely to be eligible for the trial. Practically, this will include any patient who is receiving mechanical non-invasive or invasive ventilation, and in the opinion of the responsible intensive care consultant is likely to be receiving mechanical ventilation after 48 h. While this approach will allow patients and personal consultees sufficient time to consider participation in the trial, some may inevitably receive information regarding a trial for which they (or their family member) do not become eligible.

### Withdrawal of consent

Participants will have the right to withdraw from the trial at any time for any reason, or without giving a reason. Participants no longer wishing to participate in the trial may choose either to withdraw completely, or to withdraw only from the study treatment, but continue to provide follow-up data. Participants will be asked if the data collected up to the point of withdrawal may be retained, and if they are happy, for the reason for the decision to withdraw to be recorded. Personal and professional consultees also retain the right to withdraw from the trial the participant whom they represent.

### Dissemination

The results of the trial will be presented at national and international meetings and published in peer-reviewed journals. A lay summary of the results will be available to trial participants on request. An online summary of the findings, and implications of the trial, will be offered to the intensive care patient support charity ICUsteps.

### Trial status

The trial is currently in follow-up. The first patient was recruited on 16 January 2012, and the final patient was recruited on 4 December 2014.
